# Sampling of estuarine ark clams (cockles) in estuarine soft sediment habitats, to inform fisheries management

**DOI:** 10.7717/peerj.21665

**Published:** 2026-08-24

**Authors:** Matthew David Taylor, Stephen Grant Morris, Rowan Campbell Chick

**Affiliations:** 1 NSW Department of Primary Industries and Regional Development, Post Stephens Fisheries Institute, Taylors Beach, New South Wales, Australia; 2 School of Biological Earth and Environmental Sciences, University of New South Wales, UNSW Sydney, New South Wales, Australia; 3 NSW Department of Primary Industries and Regional Development, Wollongbar Primary Industries Institute, Wollongbar, New South Wales, Australia

**Keywords:** Bivalve, Clam, Mud ark, Quadrat, Independent survey, Nested survey design

## Abstract

Cockles (ark clams) are infaunal molluscs that support important fisheries worldwide, but often occur across a complex range of strata. This creates challenges for designing independent survey programs to support fisheries management, and there is a need for additional published studies to aid development of such programs. Here, we improve the basal information available to inform the design of sampling programs for cockles, using Sydney Cockle (*Anadara trapezia*) as a case study. The combination of small quadrats and systematic searching of the quadrat area using the ‘finger dredge’ approach was highly effective. In a typical nested design (including quadrats, transects, subsites and sites), a considerable proportion of the overall variation in cockle abundance was detected at intermediate distances, with an upper limit of spatial dependence of <700 m. Approximately 77% of quadrats had all cockles visible upon initial observation (without ‘finger dredging’), meaning visible cockles provided a reasonable proxy for total (visible + buried) abundance. However, patterns were not consistent among subsites, suggesting site-specific factors may influence cockle burial. Presence of epiphytic algae (‘cockle weed’) provided a poor proxy for both presence and abundance, as algae was absent when cockles were present in 75% of observations. Analyses indicated that sampling effort should prioritise a greater number of shorter transects with fewer quadrats, allocated across a grid with <350 m centres. The patterns resolved are broadly useful for developing sampling programs for both cockles, and other infaunal bivalve molluscs.

## Introduction

Cockles or ark clams (Subfamily Anadarinae) are prominent members of many estuarine soft sediment communities. These taxa can be present across diverse estuarine habitats at very high densities, and grow to a considerable size (Squires et al., 1975). Ecologically, the functional role of these species is poorly studied. As filter feeders and primary consumers with the capacity to persist at high densities, they can display high productivity (*e.g.*,  Broom, 1983) with the potential to have a substantial influence on trophic pathways in estuarine systems.

Cockles support commercial (*e.g.*, MacKenzie, 2001) and recreational (Taylor & Chick, 2024) fisheries, and are an important aquaculture species across many countries (Borrero, 1986; Broom, 1985). In Australia, members of this taxa have formed an important food source for Australian Aboriginal peoples for many thousands of years (Catterall & Poiner, 1987), and support important subsistence fisheries in other areas of the world (*e.g.*, Tawake, Vuki & Aalbersberg, 2007). The most common form of collection is by hand gathering, although dredging is sometimes allowed (*e.g.*, Dewi et al., 2019). Cockles are thought to be comparatively sessile, and abundances are unlikely to show the tidal or diel variation in abundance or gear avoidance that is encountered when sampling other, more mobile estuarine species (*e.g.*, fish and crustaceans). Nevertheless, these taxa persist across multiple habitat strata, including shallow water not accessible by boat, deep waters not accessible by foot, vegetated and unvegetated soft sediments, muddy and sandy habitats, as well as accessible, exploited/harvested beds and less accessible, non-harvested beds. Furthermore, cockles can be clearly visible when they are on or protruding from the benthos, but can also be almost wholly buried with only the anterior margin protruding. Anadarinae, however, are generally poor burrowers and are normally only partly buried (Brotohadikusomo, 1994).

With cockles occurring across a range of strata and at varying sediment depths, quantitative sampling of these taxa is complex. Benthic infaunal species can display substantial spatial heterogeneity across habitats and environmental gradients (*e.g.*, Kraan et al., 2009; Ysebaert & Herman, 2002). However, such variability has not been systematically examined for *Anadara* spp. The resolution of abundance patterns can be strongly influenced by the sampling approach employed (Reamon et al., 2024), so efficiency and bias should ideally be evaluated under field conditions. In particular, the use of abundance proxies in place of exhaustive counts (*e.g.*, Magorrian & Service, 1998; Zhao et al., 2024), requires careful consideration of performance across sites and spatial strata. Robust indices of abundance are critical for fisheries assessments and broader ecosystem monitoring; however, resolving the suitability and performance of the methods and design selected is essential for the development of reliable longitudinal sampling programs. If sampling yields inaccurate indices, this can inflate uncertainty and reduce the reliability of stock assessments, potentially leading to ineffective management.

**Figure 1:**
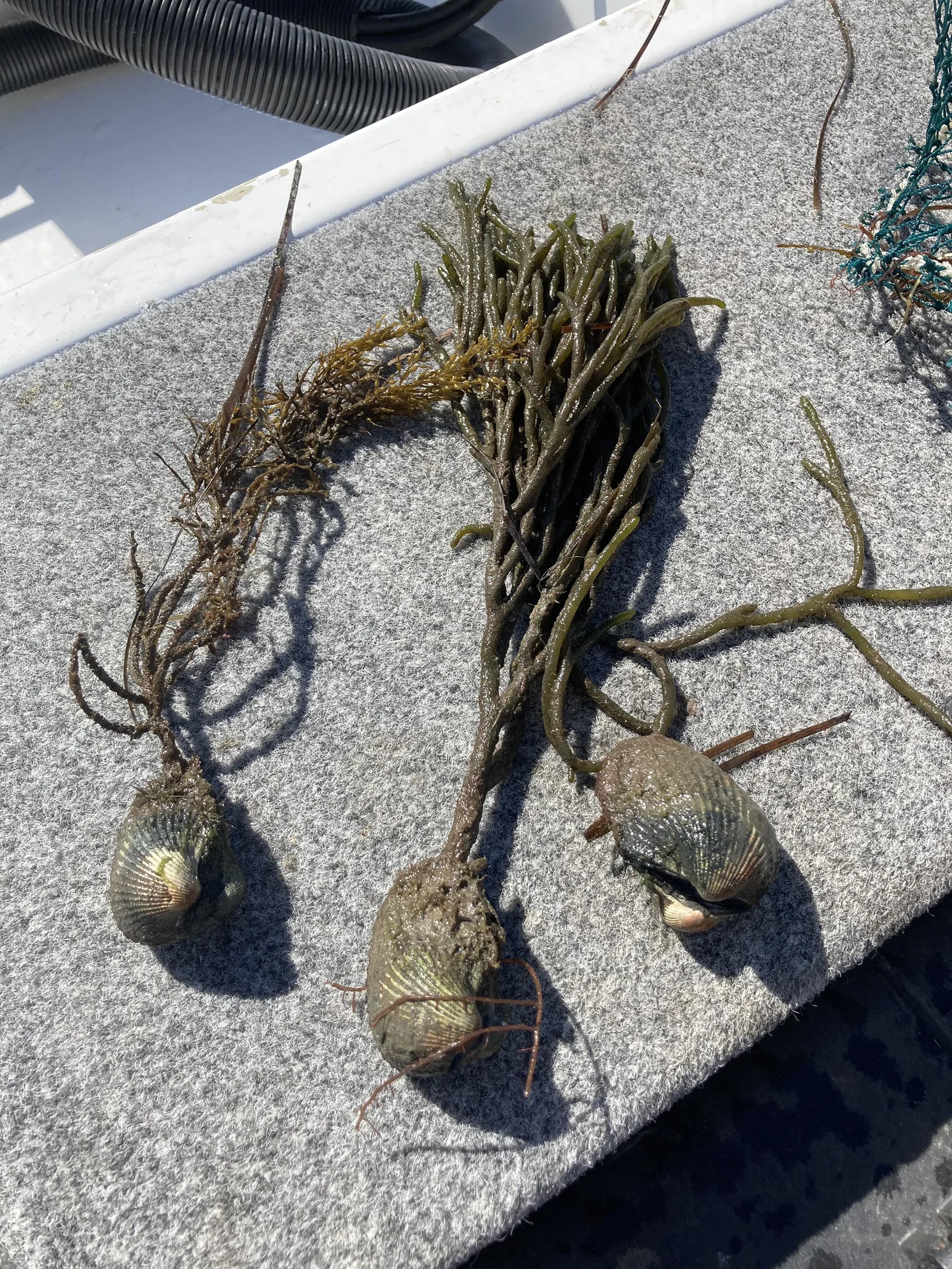
Photograph showing Sydney Cockle (*Anadara trapezia*) with epiphytic macroalgae attached. A specimen without macroalgae is included for reference (see animal on the right).

This study sought to improve the basal information available to inform the design of sampling programs for cockles, using Sydney Cockle (formerly known as the Estuary Cockle), *Anadara trapezia* as a case study. Sydney Cockle is the most common ark clam in south-eastern Australia. The species supports important commercial, recreational, and cultural fisheries in some estuaries (Taylor et al., 2026), but anecdotal evidence suggests that exploitation may have greatly increased over the past 10 years. Coupled with the comparative dearth of information on this species, this has created a need for both basal ecological research, and ongoing surveys to inform stock assessment and quota setting. Published research to date has not yet explicitly examined patterns of variation in abundance of Sydney Cockle (or other Anadarinae) from a sampling design perspective, nor have proxies of abundance (*e.g.*, relationships between visible and buried cockles) been evaluated. Specifically, we conducted sampling across multiple spatial scales to address three objectives: (1) partition sources of variability in abundance within a typical nested sampling design; (2) assess the influence of spatial separation on variability in abundance; and (3) evaluate visible cockle abundance as a potential proxy for total abundance. We hypothesised that variability in abundance would be greatest at fine spatial scales, but with additional divergence in abundance with increasing distance among sampling strata. Sydney Cockle are often colonised by epiphytic macroalgae (‘cockle weed’, Fig. 1, Inglis, 1994) which is visible above the sediment and seagrass canopy. We also examined whether the presence of this algae could serve as an indicator of cockle occurrence or relative abundance.

## Methods

### Survey location and design

**Figure 2:**
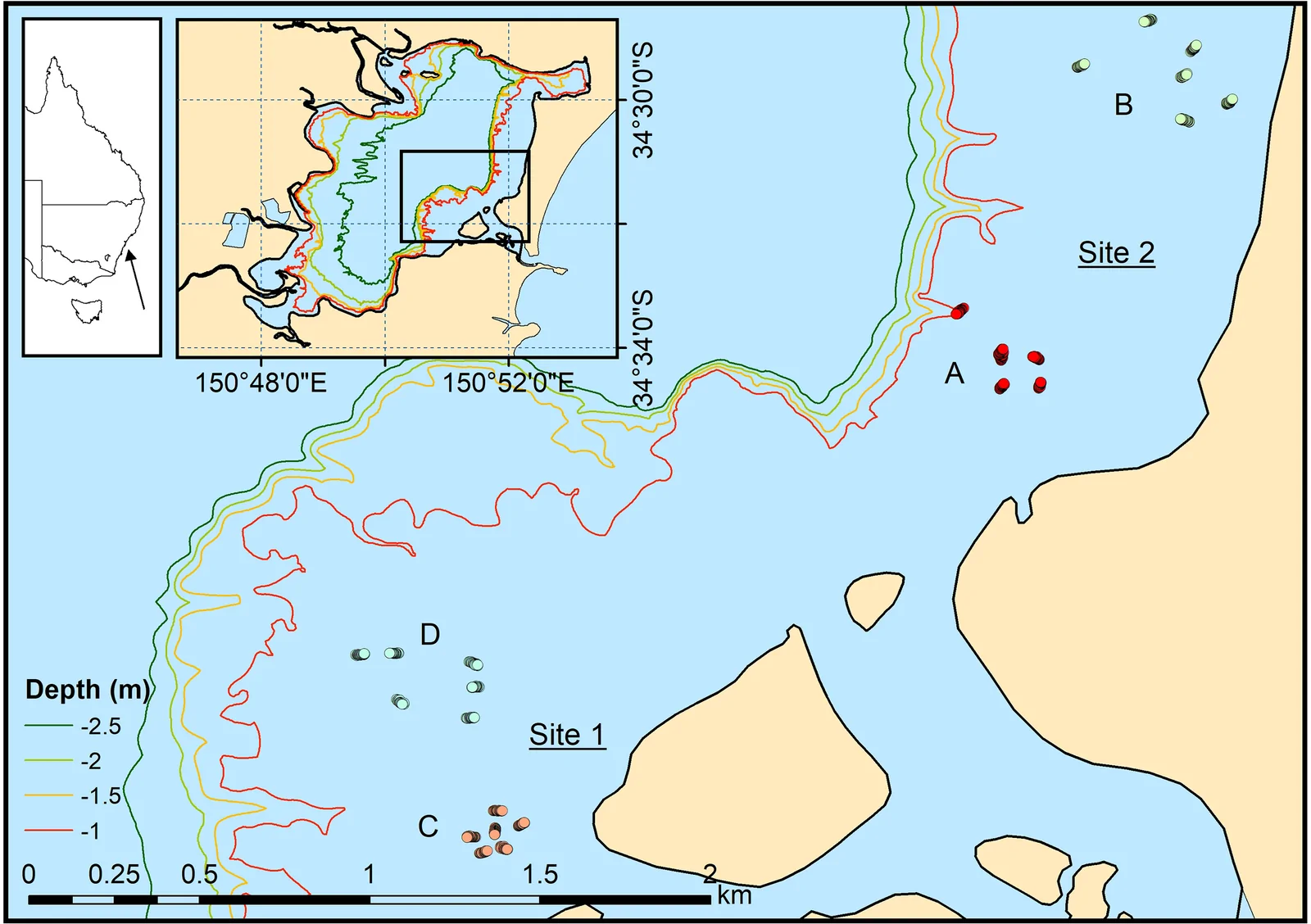
Map of study area. Map indicates the location of Lake Illawarra on the east coast of Australia (top right), the entire estuary with graticule for reference, and the site (labelled with numerals) and subsite (labelled with letters) configuration, with transects denoted as short lines of filled circles. Depth contours are also indicated.

The survey was undertaken in Lake Illawarra (34.5°S, 150.9°E; Fig. 2), a wave-dominated estuary on the south coast of New South Wales (NSW), Australia. The estuary has a waterway area of 36 km^2^, and primarily urban and agricultural catchment of area ∼240 km^2^. The average water depth is ∼2 m, and the estuary has extensive intertidal and shallow subtidal sand/mud flats and seagrass beds around its perimeter. The estuary is commercially fished, experiences substantial recreational fishing for the species, and has been a site of Aboriginal cultural fishing for thousands of years (Bursill, Donaldson & Jacobs, 2015).

**Figure 3:**
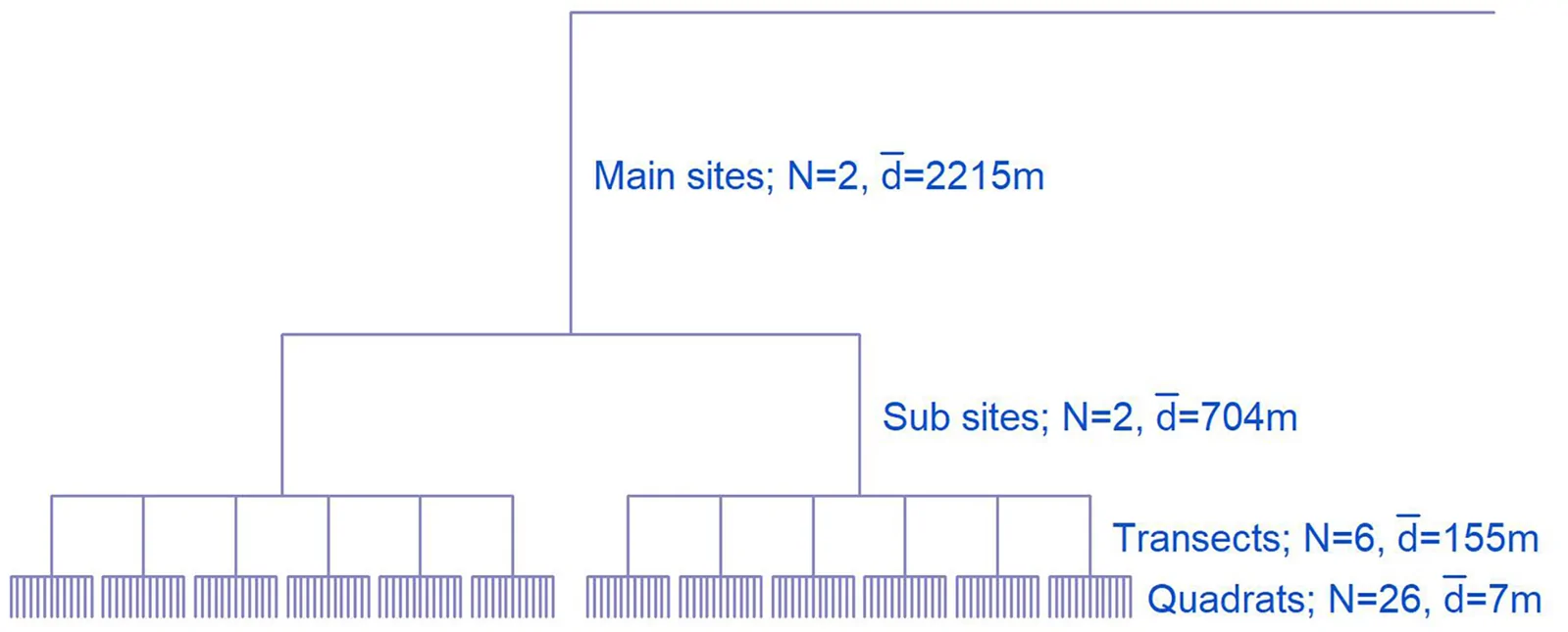
Schematic of the survey design. Diagram shows the stratum labels, number of sampling units within each stratum (N) and mean distance between sampling unit centres (d).

The nested survey design (Fig. 3) included the strata *main site* (*n* = 2), *subsite* (*n* = 4), *transect* (*n* = 24, 20 m long) and *quadrat position* (*n* = 480, 0.3 × 0.3 m), with 20 quadrat positions sampled along each transect (at 1-m increments). In addition, three quadrat positions were randomly selected within each transect, and for these positions three adjacent (side-by-side) quadrats were sampled. With these additional quadrats, there were nominally 26 quadrats undertaken per transect, although for one transect only two adjacent (side-by-side) quadrats were sampled instead of three (subsite C, transect 3). All transects were in ≤1.3 m of water.

### Sampling methodology

The transect starting location was randomly chosen within each subsite, as was the bearing that the transect followed. For each transect, a weighted rope with marked 1-metre increments was deployed (moored with a weight at the start and the end), commencing at the starting point and continuing along the random bearing. The quadrat was deployed on alternate sides of the rope at each increment, except for those quadrat positions that were randomly selected for adjacent quadrats.

Once a transect was set up and any suspended sediment in the water column had cleared, a snorkeller entered the water and proceeded to work through the quadrat sequence. For each quadrat, an initial visual search quantified the number of individuals for which any part of the body was observed at the surface of the sediment. These were recorded as ‘visible’ cockles, and removed from the quadrat. Next, the area inside the quadrat was subjected to a full search and dredged by hand to the depth of ∼10 cm (‘finger dredge’), and any remaining individuals recorded as ‘non-visible’ abundance. Individual cockles were assessed for the presence of attached cockle weed and their shell length measured (the longest distance from the rear margin to the front margin, in millimetres) using vernier callipers. Animals were then returned to water, away from the transect.

Sample collection was permitted under s37 permit P01/0059 issued by the NSW Department of Primary Industries and Regional Development. The research was performed under NSW Department of Primary Industries and Regional Development (Fisheries) Animal Research Authority #0410, and complied with the New South Wales *Animal Research Act (1985)*. While there are no taxon specific guidelines for working with bivalve molluscs, this research followed the general guidelines for animal care outlined in the Australian Code for the Care and Use of Animals for Scientific Purposes. This investigation also took all measures possible to follow the principles of Replacement, Reduction and Refinement, including returning animals to the water alive and in good condition, near the location where they were captured. Furthermore, the principal intention of the study was to reduce and refine animal use in future studies through optimisation of survey design for *Anadara* spp.

### Data analysis

The spatial scale of variation in cockle count per quadrat was portrayed by a variogram based on distance between the survey strata, as described in Webster et al. (2006). A generalised linear mixed model (GLMM) was constructed (Eq. 1) to estimate components of variance in each stratum of the survey (Fig. 3).

(1) \begin{eqnarray*}\log \nolimits ({Y}_{ijkl})=\mu +{M}_{i}+{S}_{ij}+{T}_{ijk}+{Q}_{ijkl}\end{eqnarray*}

where log(*Y*_*ijkl*_) is the natural logarithm of the expected number of cockles captured in quadrat (*Q*) *l* within transect (T) *k* in subsite (S) *j* in main site (M) *i*, and µ is the survey mean count per quadrat (log scale). The GLMM was assumed to be robust to the slightly unbalanced design created from the missingness of two adjacent quadrats, outlined earlier. The observed counts per quadrat were assumed to be drawn from a Poisson distribution and the variables M, S, T and Q were assumed to be random samples drawn from normal distributions with mean zero and variances ${\sigma }_{M}^{2},{\sigma }_{S}^{2},{\sigma }_{T}^{2},{\sigma }_{Q}^{2}$ respectively. As shown in Webster et al. (2006) an approximate variogram can be calculated by plotting the cumulated percentage of total variance (${\sigma }^{2}={\sigma }_{M}^{2}+{\sigma }_{S}^{2}+{\sigma }_{T}^{2}+{\sigma }_{Q}^{2}$) for each stratum against the average distance between sampling unit centres (Fig. 3). A model allowing for the nesting of 3 quadrats within 3 positions in each transect was tested but gave no improvement based on the Akaike information criterion. A Monte-Carlo simulation study was used to demonstrate that the observed data was no more dispersed (*p* = 0.7) than expected under the fitted model (Hartig, 2024).

The return on time invested in a full search to account for non-visible cockles was evaluated by estimating cockle density at each subsite based on visible and total cockles per quadrat. Estimates of mean density in each subsite were obtained from GLMMs similar to Eq. 1 but treating subsites as fixed effects. The average size of cockle at each subsite was estimated by linear mixed model treating subsites as fixed effects, transects as random effects and assuming a gaussian error distribution. All data analysis and graphical presentation was conducted in the R environment (R Core Team, 2025) with particular use of the lme4 package (Bates et al., 2015) for model fit and DHARMa package (Hartig, 2024) for diagnostics. Full analysis code and data files are available at https://doi.org/10.5281/zenodo.19341874.

## Results

### General observations

Overall, the 622 quadrats completed yielded 516 cockles, which equated to a global average density across the study of 9.2 ind m^−2^ (∼0.83 cockles per quadrat). Benthic habitats encountered included unvegetated soft sediment, as well as beds of *Zostera*, *Ruppia* and *Halophila*, with the majority of quadrats encompassing some type of vegetation. Water quality conditions during the trial survey ranged between 19–26 °C, 34.2–34.7 salinity, and 6.7–9.9 mg L^−1^ dissolved oxygen.

### Spatial variation

**Figure 4:**
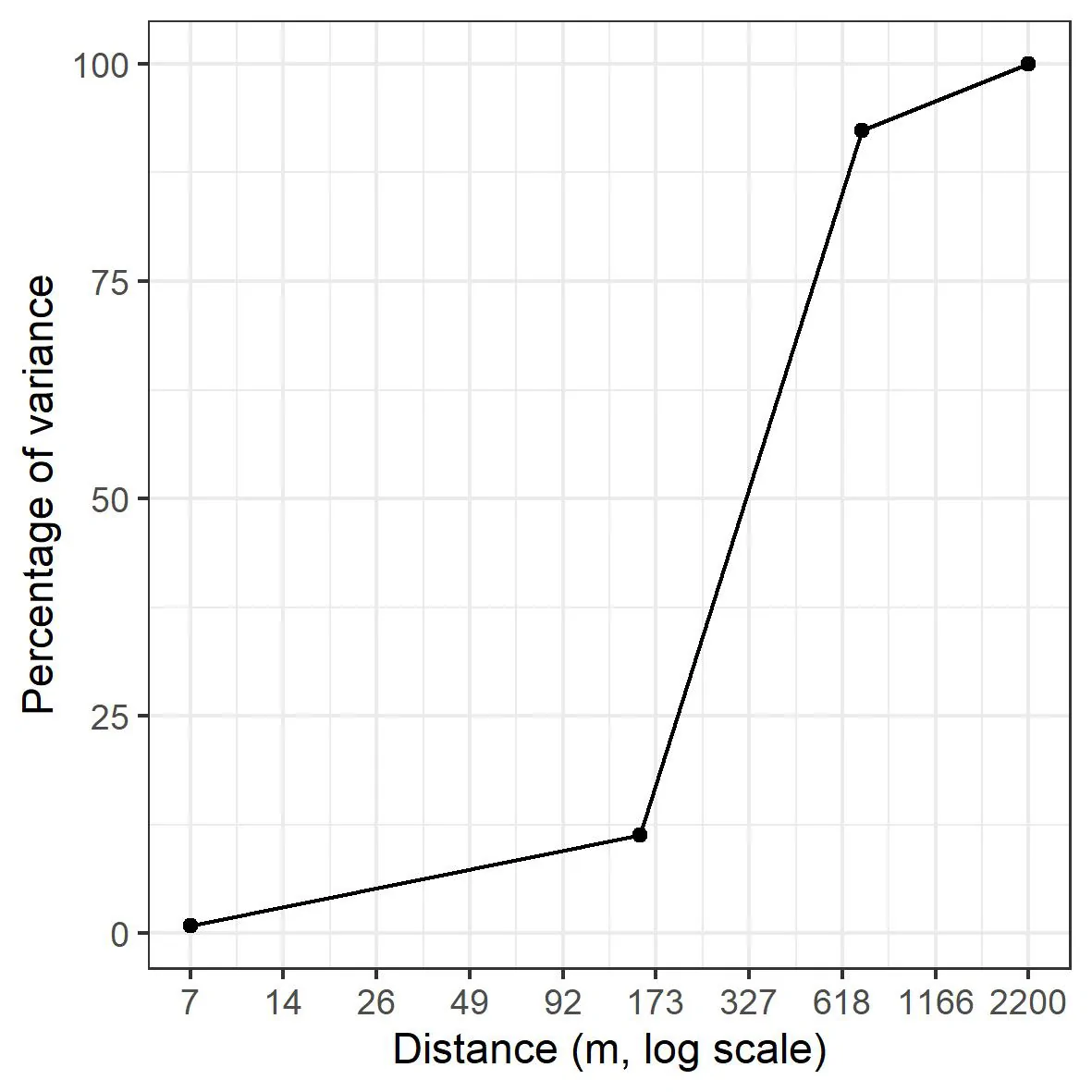
Sample variogram of cockle counts per quadrat.

A low level of sample variation was associated with quadrats (0.9%, ${\sigma }_{Q}^{2}=0.04$), transects (10.4%, ${\sigma }_{T}^{2}=0.52$) and main sites (7.7%, ${\sigma }_{M}^{2}=0.38$), with the majority of total variance (81%, ${\sigma }_{S}^{2}=4.00$) in cockle abundance occurring over intermediate distances (*i.e.,* ‘subsites’) in our nested design. These patterns were also evident in the sample variogram, which indicated that spatial dependence was largely captured within a distance of ∼700 m, where approximately 90% of the total variance was explained (Fig. 4).

### Proxies for abundance

**Figure 5:**
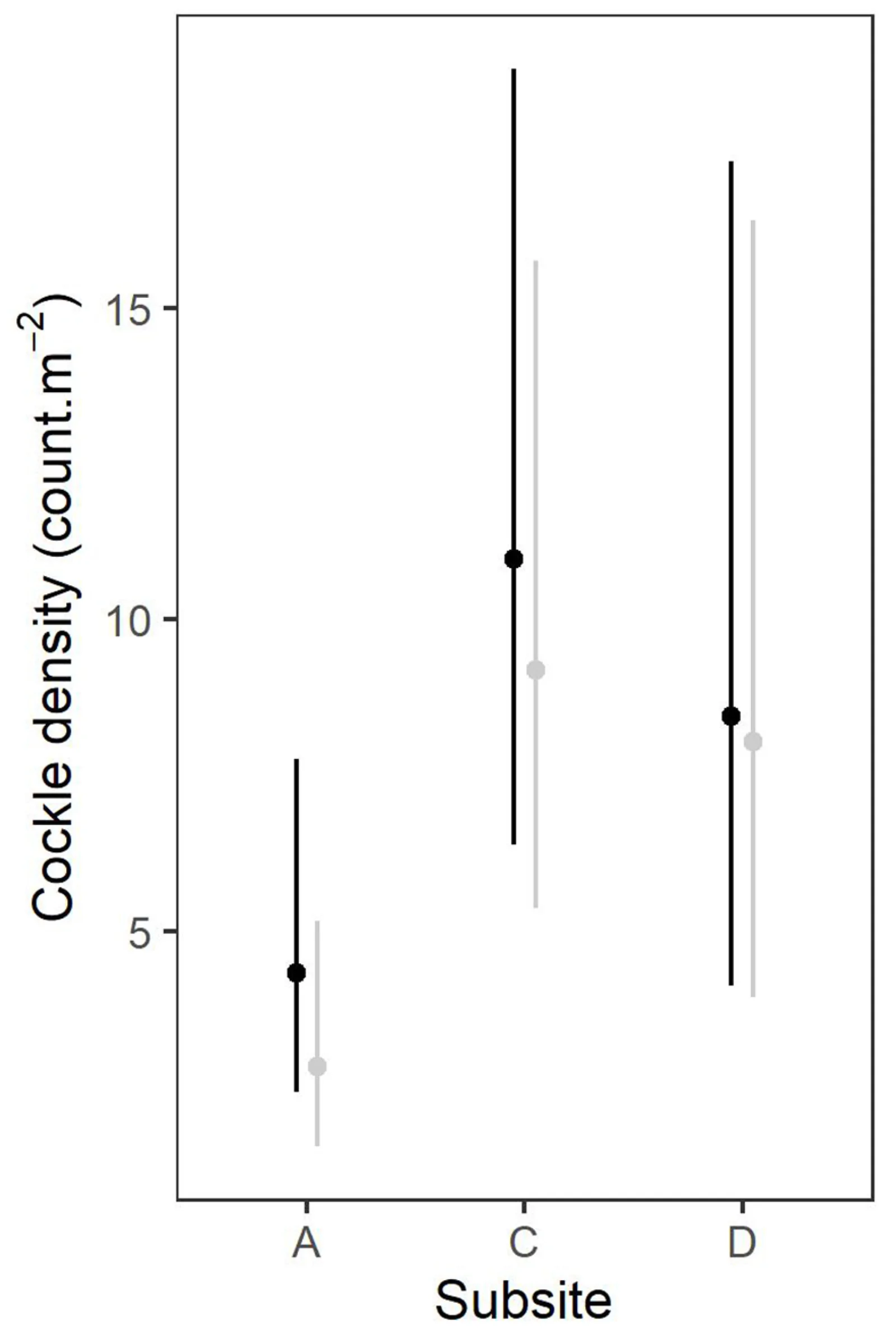
Average density for visible and total cockles encountered across subsites (A, C) and (D). Vertical bars span 95% confidence intervals.

Counts of visible cockles could not be reliably completed in quadrats which contained dense seagrass, and consequently 75 quadrats (all were from subsite D) were excluded from comparison between ‘visible’ and ‘total’ cockle counts. For the remaining quadrats, it was found that only 16% of the individuals collected were classified as non-visible, with 84% of cockles visible upon the initial visual search (Table 1). These visible cockles provided a reasonable proxy for total cockle abundance, as 77% of quadrats with cockles present had all cockles visible upon initial observation (Table 1). Comparison of estimated density in each subsite between the visible and total count data did show some deviation among sites (Fig. 5), with a proportionally lower mean density for subsite A when only visible cockles were considered (although confidence limits did overlap between the two data sets for all subsites). There was no major difference in mean size evident among subsites (Table 2).

**Table 1:** Number and percentage of quadrats and cockles, classified by cockle position. Data excludes 75 quadrats with very dense seagrass.

Cockle location	Quadrats	Cockles
All visible	159 (29.1%)	257 (74.5%)
All non-visible	26 (4.8%)	30 (8.7%)
Both visible and non-visible	21 (3.8%)	58 (16.8% = 9.3% visible + 7.5% non-visible)
No cockles present	341 (62.3%)	

The presence of epiphytic algae provided a comparatively poor proxy for cockle abundance, with epiphytic algae absent when cockles were present in ∼75% of cases. By treating presence of algae as a test for presence of cockles in a quadrat in comparison with true presence detected by finger dredging, a confusion matrix yielded a test sensitivity of 0.25. Only 60 individuals (11% of all cockles sampled in the survey) had weed attached, and cockles with attached weed were only present in 8.5% of quadrats.

## Discussion

The combination of small quadrats and systematic searching of the quadrat area using the finger dredge was highly effective, with rechecking of random quadrats throughout the study suggesting a very high sampling efficiency. Quadrat searches could also be conducted comparatively quickly, with quadrats in seagrass or macrophyte habitats generally taking no longer than a minute to search. The results suggest that much of the variance in cockle abundance was driven by patchiness in distribution, such that patches are detected at greater distances whereas neighbouring quadrats tended to be similar in presence or absence of individuals.

### Spatial variability and contributing factors

In relation to our original hypotheses of strong small-scale patchiness and increasing divergence across larger distances, abundance was found to vary mostly across intermediate scales. Much of the observed variation actually occurred within contiguous beds, and the semi-variance rapidly accumulated across spatial scales that nested wholly within subsites. While no directly comparable surveys were identified for *Anadara* spp., this pattern is similar to outcomes from hierarchical surveys for other cockle species, which have reported peaks in variability across scales of hundreds of metres (*e.g.*, Clemente & Thomsen, 2024; Meyer et al., 2021). Previous work on abundance patterns in *Anadara* spp. does however highlight a range of variables that contribute to spatial variation, including fishing pressure, sedimentary composition, habitat, spatially variable recruitment and settlement driven by estuarine hydrology, and underlying environmental gradients. Silina (2006) found fishing pressure to be the proximal factor influencing abundance of *Anadara broughtoni*, but also noted salinity and suspended particulate matter as drivers of abundance. Ramadhan, Pursetyo & Dewi (2019) highlighted the influence of various water quality variables on the abundance of *Anadara* sp., whereas Strafella et al. (2017) noted sediment characteristics as the most important driver. For Sydney Cockle, Gribben & Wright (2006) showed that macrophytes were important in driving abundance of young cockles, with the highest densities occurring in sparsely vegetated beds of invasive seaweed.

**Table 2:** Total capture and average length (SE) by subsite.

Subsite	Total capture	Length (mm)
A	85	47.6 (1.8)
B	1	55.7^*^
C	175	47.2 (1.4)
D	255	47.2 (1.4)

**Notes.**

* Single observation.

Further to Silina (2006), more recent work highlights the impact of harvesting and its potential influence on abundance (Aswani, Flores & Broitman, 2015; Beukema & Dekker, 2018). We can only speculate on the impact of fishing on the patterns observed in our study, as no data on targeted fishing effort are available. Subsite B was characterised by almost no cockles, despite being at similar depths and on the same contiguous sand flat as subsite A. Subsite B is easier to access by foot and is adjacent to a foreshore park, and may thus be more accessible to hand gatherers, which raises the possibility of fishing impacting abundance at this location. However, reliable information on small-scale patterns in fishing effort are required to properly account for this. Such data may provide a useful covariate to aid interpretation of patterns in abundance in future studies.

Much of the literature highlights the importance of abiotic environmental gradients in structuring the distribution and abundance of sedimentary bivalves in estuarine systems. Evaluation of nested sampling designs in shallow coastal lakes, however, provides novel insight into the components of spatial variation in bivalve abundance under conditions where strong environmental gradients are less influential. Coastal lakes are typically shallow and experience strong wind- and tide-driven mixing (Kjerfve & Magill, 1989), which can reduce stratification. Coupled with limited freshwater input, gradients in water quality may therefore be weaker than in more riverine estuaries (Roy et al., 2001), potentially decreasing their overall influence on cockles and other benthic fauna. Consequently, our results are likely to be more influenced by other drivers of abundance, such as habitat and sediment characteristics, and the influence of hydrology and circulation on recruitment and settlement. These represent important variables to consider in future surveys, particularly where environmental gradients are likely to be weak and spatial variation in abundance may be more heavily driven by local-scale processes.

### Recommendations for future surveys

The variogram analysis supports a clear recommendation on the spatial arrangement of sampling units—under the guideline to focus sampling at intervals of less than half the variogram range (Kerry & Oliver, 2004), spatial resolution could be captured by setting shorter transects with fewer quadrats on a grid with <350 m centres. We also note that our design provides an efficient approach to refining the spatial arrangement of sampling units, as employment of a nested sampling structure can produce an approximate variogram in exchange for modest effort compared to grid-based sampling (Webster et al., 2006).

The sampling locations selected should also be representative of the various strata of interest, as well as the life-history stage being investigated. Considering variables that are known or suspected to influence abundance (outlined above) will be important if patterns are to be compared across broader spatial scales. While there is potential for seagrass and macrophytes to impact abundance of Sydney Cockle (*e.g.*, Gribben & Wright, 2006), incorporating these variables in nested longitudinal survey designs may prove challenging. Some of the main species of seagrass (*e.g.*, *Zostera*, *Halophila* and *Ruppia*) encountered in southeastern Australian estuaries display substantial seasonal and inter-annual variability (West & Glasby, 2022). For this reason, it may be best to incorporate seagrass-related variables as covariates in long-term longitudinal surveys, rather than as a specific habitat class.

### Suitability of proxies of abundance

While often employed in surveys of benthic macrofauna (*e.g.*, Magorrian & Service, 1998; Selin, 2011; Zhao et al., 2024), abundance proxies are rarely validated. This is a notable limitation, as proxies can be influenced by animal behaviour and site-specific habitat characteristics, which means that relationships between observed and actual densities may be inconsistent across spatiotemporal scales. For bivalves, the performance of proxy measures (*e.g.*, visual, video-based, and other indicators like cockle weed) may vary among habitats and conditions, highlighting the importance of quantitative validation. For example, visual surveys of the mussel *Unio crassus* detected only ∼10% of individuals present, and full excavation was required for informative data (Lamand & Beisel, 2014). In our study, the presence of cockle weed provided neither an indicator of abundance, nor even a useful indicator of presence for Sydney Cockle. This was possibly due to local-scale influences on settlement and mortality of algal epiphytes on cockle shells (Inglis, 1994).

While visible animals provided an informative proxy for Sydney Cockle, variable deviations between visible search and finger dredge counts suggests that there may be site-specific factors that influence the extent of cockle burial. Similar size compositions among sites indicates that this was unlikely to be a size-related effect, but other variables such as water quality are known to impact position in the sediment in Sydney Cockle (Wright et al., 2010). Coupled with the effect of dense seagrass on visible counts encountered at subsite D, relying on counts of visible cockles should be applied with caution, and may be most appropriate as site-specific abundance indices, rather than to support comparison across spatial strata. Careful validation of proxy measures is essential to ensure that they provide reliable and consistent estimates across spatial and temporal scales.

## Conclusion

Abundance surveys are essential for robust resource assessment, and are especially important for informing harvest strategies for bivalve species (Gorman et al., 2011). In addition, sedimentary bivalves integrate conditions in the surrounding environment and are widely used as bioindicators of water quality and pollution (Ochoa-Esteso et al., 2024; Vaughn & Hoellein, 2018). Estimation of robust abundance indices is thus also critical for monitoring ecosystem health. However, efficacious sampling requires consideration of variance components across relevant spatial strata such that sampling effort can be appropriately targeted. Pilot studies are important for quantifying the extent of variation across different spatial strata, and while they provide valuable guidance for designing larger longitudinal surveys, such preliminary work is infrequently published (Andrew & Mapstone, 1987). Our results provide a quantitative basis to support development of future surveys for Sydney Cockle, but are also broadly applicable to other similar taxa. Specifically, (1) Use of quadrats and the finger dredge is highly efficient for capturing cockles, and large numbers of quadrats can be completed in a comparatively short period of time, even within seagrass habitats; (2) the presence of epiphytic macroalgae is a poor indicator for cockle presence or abundance; (3) visible cockles provide a reasonable proxy for total cockle abundance, however site-specific attributes may have a confounding effect on this; (4) when planning longitudinal surveys for cockles, sampling effort should prioritise a greater number of shorter transects with fewer quadrats. These findings provide useful information to aid the design of efficient and robust monitoring programs for sedimentary bivalves.

## Additional Information and Declarations

### Field Study Permissions

The following information was supplied relating to field study approvals (i.e., approving body and any reference numbers): Sample collection was permitted under s37 permit P01/0059 issued by the NSW Department of Primary Industries and Regional Development.

### Data Availability

The following information was supplied regarding data availability: The data and R-code are available at Zenodo: Morris, S. (2026). Sampling of estuarine ark clams (cockles) in estuarine soft sediment habitats, to inform fisheries management [Dataset]. Zenodo. https://doi.org/10.5281/zenodo.20652010.
